# Two isostructural 3-(5-ar­yloxy-3-methyl-1-phenyl-1*H*-pyrazol-4-yl)-1-(thio­phen-2-yl)prop-2-en-1-ones: disorder and supra­molecular assembly

**DOI:** 10.1107/S205698901901658X

**Published:** 2020-01-01

**Authors:** Mohammed A. E. Shaibah, Hemmige S. Yathirajan, Nagaraj Manju, Balakrishna Kalluraya, Ravindranath S. Rathore, Christopher Glidewell

**Affiliations:** a Department of Studies in Chemistry, University of Mysore, Manasagangotri, Mysuru-570 006, India; b Department of Studies in Chemistry, Mangalore University, Mangalagangotri, Mangalore-574 199, India; c Department of Bioinformatics, School of Earth, Biological and Environmental Sciences, Central University of South Bihar, Gaya-824 236, India; d School of Chemistry, University of St Andrews, St Andrews, Fife KY16 9ST, UK

**Keywords:** heterocyclic compounds, pyrazoles, crystal structure, disorder, mol­ecular conformation, hydrogen bonding, supra­molecular assembly

## Abstract

Two new chalcones containing both pyrazole and thio­phene substituents have been prepared and structurally characterized. 3-(3-Methyl-5-phen­oxy-1-phenyl-1*H*-pyrazol-4-yl)-1-(thio­phen-2-yl)prop-2-en-1-one, C_23_H_18_N_2_O_2_S (I), and 3-[3-methyl-5-(2-methyl­phen­oxy)-1-phenyl-1*H*-pyrazol-4-yl]-1-(thio­phen-2-yl)prop-2-en-1-one, C_24_H_20_N_2_O_2_S (II), are isomorphous as well as isostructural, and in each the thio­phene substituent is disordered over two sets of atomic sites having occupancies 0.844 (3) and 0.156 (3) in (I), and 0.883 (2) and 0.117 (2) in (II). In each structure, the mol­ecules are linked into sheets by a combination of C—H⋯N and C—H⋯O hydrogen bonds. Comparisons are made with some related compounds.

## Abstract 2

In each of two isostructural 3-(5-ar­yloxy-3-methyl-1-phenyl-1*H*-pyrazol-4-yl)-1-(thio­phen-2-yl)prop-2-en-1-ones, the thio­phene unit is disordered over two sets of atomic sites and a combination of C—H⋯N and C—H⋯O hydrogen bonds link the mol­ecules into sheets.

## Chemical context   

Pyrazole derivatives exhibit a wide range of pharmacological activity (Karrouchi *et al.*, 2018 ▸), including analgesic (Vijesh *et al.*, 2013 ▸), anti­cancer (Dawood *et al.*, 2013 ▸; Koca *et al.*, 2013 ▸), anti­depressant (Mathew *et al.*, 2014 ▸), anti­fungal (Zhang *et al.*, 2017 ▸), anti-inflammatory (Badawey & El-Ashmawey, 1998 ▸) and anti­microbial (Vijesh *et al.*, 2013 ▸) activities. In addition, a range of thio­phene-based heterocyclic compounds have been shown to exhibit anti­microbial activity (Mabkhot *et al.*, 2016 ▸).
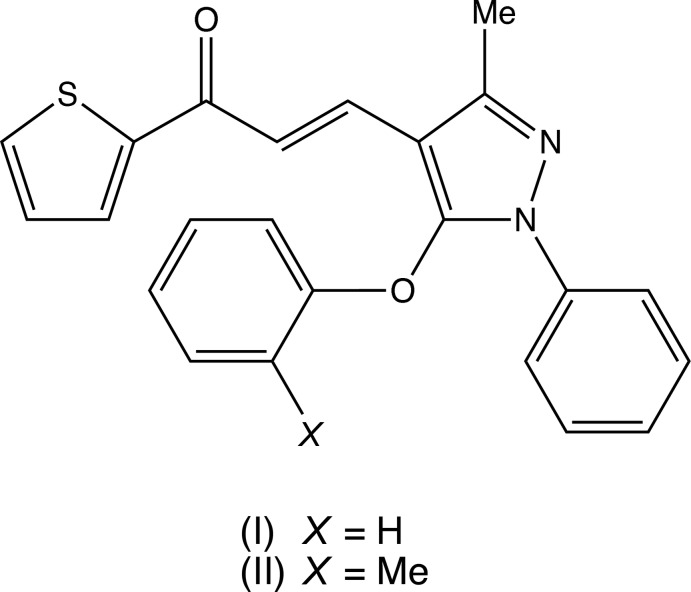


**Figure 1:**
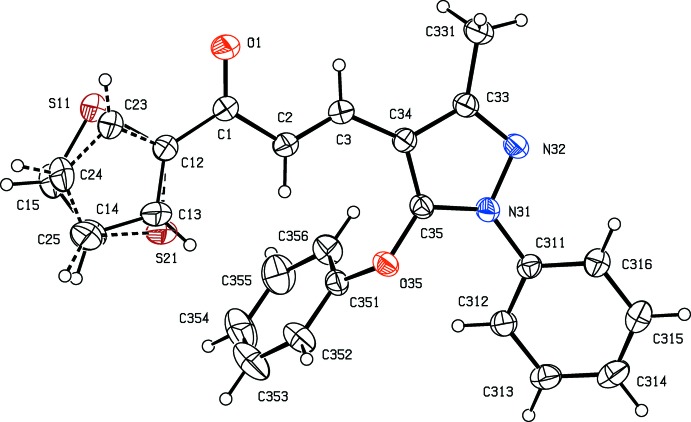
The mol­ecular structure of compound (I), showing the atom-labelling scheme, and the disorder in the thio­phen-2-yl substituent, where the major disorder component has been drawn using full lines and the minor disorder component has been drawn using dashed lines.

**Figure 2:**
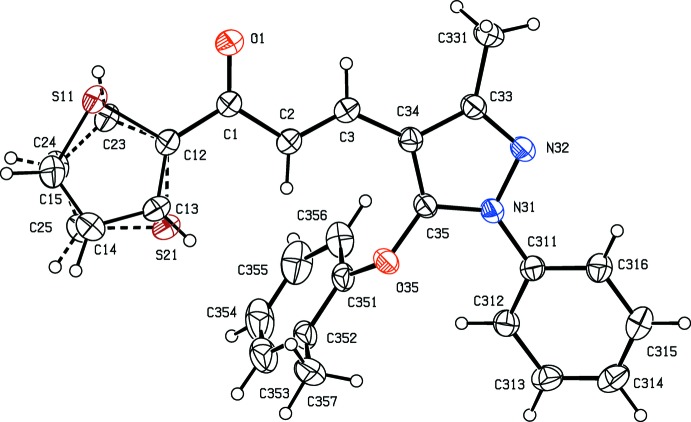
The mol­ecular structure of compound (II), showing the atom-labelling scheme, and the disorder in the thio­phen-2-yl substituent, where the major disorder component has been drawn using full lines and the minor disorder component has been drawn using dashed lines.

With these observations in mind, we have now synthesized two new chalcones containing both pyrazole and thio­phene moieties, namely 3-(3-methyl-5-phen­oxy-1-phenyl-1*H*-pyrazol-4-yl)-1-(thio­phen-2-yl)prop-2-en-1-one, C_23_H_18_N_2_O_2_S (I) (Fig. 1 ▸), and 3-[3-methyl-5-(2-methyl­phen­oxy)-1-phenyl-1*H*-pyrazol-4-yl]-1-(thio­phen-2-yl)prop-2-en-1-one, C_24_H_20_N_2_O_2_S (II) (Fig. 2 ▸), and here we report their mol­ecular and supra­molecular structures.

## Structural commentary   

Compounds (I) and (II) are isomorphous with unit-cell volumes which differ by only *ca* 1% and, with appropriate adjustment of the substituent at atom C352 (H versus CH_3_), each structure can be smoothly refined using the atomic coordinates of the other as the starting point.

In each structure, the thienyl group is disordered over two sets of atomic sites having occupancies 0.844 (3) and 0.156 (3) in (I), and 0.883 (2) and 0.117 (2) in (II): in each case, the two disorder components are approximately related by a rotation of *ca* 180° about the C1—C12 bond (Figs. 1 ▸ and 2 ▸). It is by no means clear why the occupancies of the two disorder components in each compound are so different, particularly as the two disorder components form similar inter­molecular hydrogen bonds (Section 3).

For both compounds, the central space unit between atoms C12 and C34, the pyrazole ring and the major disorder component of the thienyl ring are almost coplanar, and the r.m.s. deviations of the atoms from the mean planes through these units are only 0.055 Å in (I) and 0.102 Å in (II). By contrast, the two pendent aryl rings are markedly displaced from this plane: the dihedral angles between the pyrazole ring and the rings (C311–C316) and (C351–C356) are 29.99 (11) and 78.60 (6)°, respectively, in (I), and 27.90 (11) and 81.13 (6)° in (II). On the other hand, atom C35 is, in each structure, displaced from the plane (O35/C351–C356) by only 0.097 (3) Å in (I) and 0.017 (3) Å in (II). Associated with this near co-planarity, the two exocyclic C—C—O angles at atom C351 differ in each structure by *ca* 9°, as typically found in planar alk­oxy­arenes (Seip & Seip, 1973 ▸; Ferguson *et al.*, 1996 ▸).

## Supra­molecular features   

**Figure 3:**
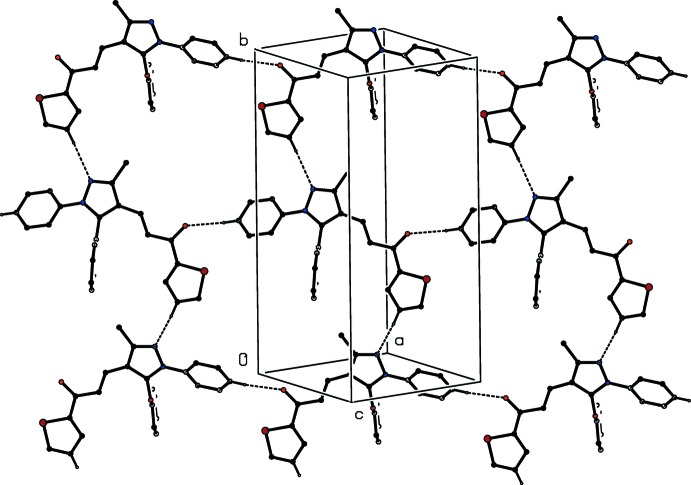
Part of the crystal structure of compound (I) showing the formation of a hydrogen-bonded sheet lying parallel to (10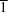). Hydrogen bonds are drawn as dashed lines and, for the sake of clarity, the minor disorder component and the H atoms which are not involved in the motifs shown have been omitted.

The supra­molecular assembly of compound (I) depends upon just two hydrogen bonds, one each of C—H⋯N and C—H⋯O types (Table 1 ▸). The C—H⋯O hydrogen bonds links mol­ecules which are related by translation to form a *C*(12) (Etter, 1990 ▸; Etter *et al.*, 1990 ▸; Bernstein *et al.*, 1995 ▸) chain running parallel to the [101] direction (Fig. 3 ▸). The C—H⋯N hydrogen bond links mol­ecules which are related by the 2_1_ screw axis along (0.5, *y*, 0.25) to form a *C*(10) chain running parallel to the [010] direction (Fig. 3 ▸). The chain formation along [010] is independent of the disorder, since both atom C14 in the major disorder component and atom C25 in the minor component (*cf*. Fig. 1 ▸) form similar C—H⋯N hydrogen bonds. The combination of these two chain motifs generates a sheet in the form of a (4,4) net (Batten & Robson, 1998 ▸) built from 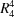
(35) rings and lying parallel to (10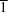). The supra­molecular assembly of compound (II) is entirely similar to that in (I), although the C—H⋯N hydrogen bond formed by the minor disorder component is rather long (Table 2 ▸).

In view of the similarities in the hydrogen bonds formed by (I) and (II), and their similar mol­ecular conformations (see Section 2), these isomorphous compounds can be described as isostructural, although it is not always the case that isomorphous pairs are strictly isostructural (Bowes *et al.*, 2003 ▸; Acosta *et al.*, 2009 ▸; Blanco *et al.*, 2012 ▸).

## Database survey   

It is of inter­est to briefly compare the structures of compounds (I) and (II) reported here with those of some related compounds. 2,5-Bis[(3,5-di­methyl­pyrazol-1-yl)carbon­yl]thio­phene (III) crystallizes with *Z*′ = 2 in space group *P*2_1_/*m* (Guzei *et al.*, 2009 ▸): the two independent mol­ecules are weakly linked by a C—H⋯O hydrogen bond but the only other direction-specific inter­actions between the mol­ecules are π–π inter­actions involving inversion-related pairs of pyrazole rings. In contrast to the simplicity of the mol­ecular constitution of (III) above, in most other structures containing both pyrazole and thio­phene units, at least one of the rings is fused. In 3,6-dimethyl-1-phenyl-4-(thio­phen-2-yl)-8-(thio­phen-2-yl­methyl­ene)-5,6,7,8- tetra­hydro-1*H*-pyrazolo­[3,4-*b*][1,6]naphthyridine (IV) (Peng *et al.*, 2009 ▸), the mol­ecules are linked into *C*(11) chains by means of C—H⋯N hydrogen bonds. The mol­ecules of 2-(3,4-dimethyl-5,5-dioxo-2*H*,4*H*-pyrazolo­[4,3-*c*][1,2]benzo­thia­zin-2-yl)-*N*′-(thio­phen-2-yl­methyl­idene)acetohydrazide (V) (Ahmad *et al.*, 2010 ▸) are linked by a combination of N—H⋯O and C—H⋯N hydrogen bonds: although the resulting aggregation was described as consisting of dimers, the mol­ecules are, in fact, linked into chains of rings, as clearly illus­trated in the original report. A chain of rings, built from a combination of N—H⋯N and C—H⋯N hydrogen bonds is also found in the structure of (*Z*)-ethyl 2-cyano-2-{2-[5,6-dimethyl-4-(thio­phen-2-yl)-1*H*-pyrazolo­[3,4-*b*]pyridin-3-yl]hydrazinyl­idene}acetate (VI) (Fun *et al.*, 2011 ▸).

In 9-(thio­phen-2-yl)-8,9-di­hydro-3*H*-pyrazolo­[4,3-*f*]quinolin-7(6*H*)-one ethanol monosolvate (VII) (Peng & Jia, 2012 ▸), the thio­phene ring is disordered over two sets of atomic sites having unequal occupancies, 0.692 (7) and 0.308 (7), much as found here for compounds (I) and (II). The mol­ecular components in (VII) are linked by N—H⋯O and O—H⋯N hydrogen bonds to form a complex chain of rings. The thio­phene ring in 5,6-dimethyl-4-(thio­phen-2-yl)-1–pyrazolo­[3,4-*b*]pyridin-3-amine (VIII) (Abdel-Aziz *et al.*, 2012 ▸) is also disordered, with occupancies of 0.777 (4) and 0.223 (4), and the mol­ecules are again linked into a chain of rings, this time by two independent N—H⋯N hydrogen bonds. Finally, we note that in [4-(2-meth­oxy­phen­yl)-3-methyl-1-phenyl-6-tri­fluoro­methyl-1*H*-pyrazolo­[3,4-*b*]pyridin-5-yl](thio­phen-2-yl)methanone (IX) (Rajni Swamy *et al.*, 2014 ▸), where the thio­phene ring is fully ordered, there are no significant hydrogen bonds of any kind.

## Synthesis and crystallization   

**Figure 4:**
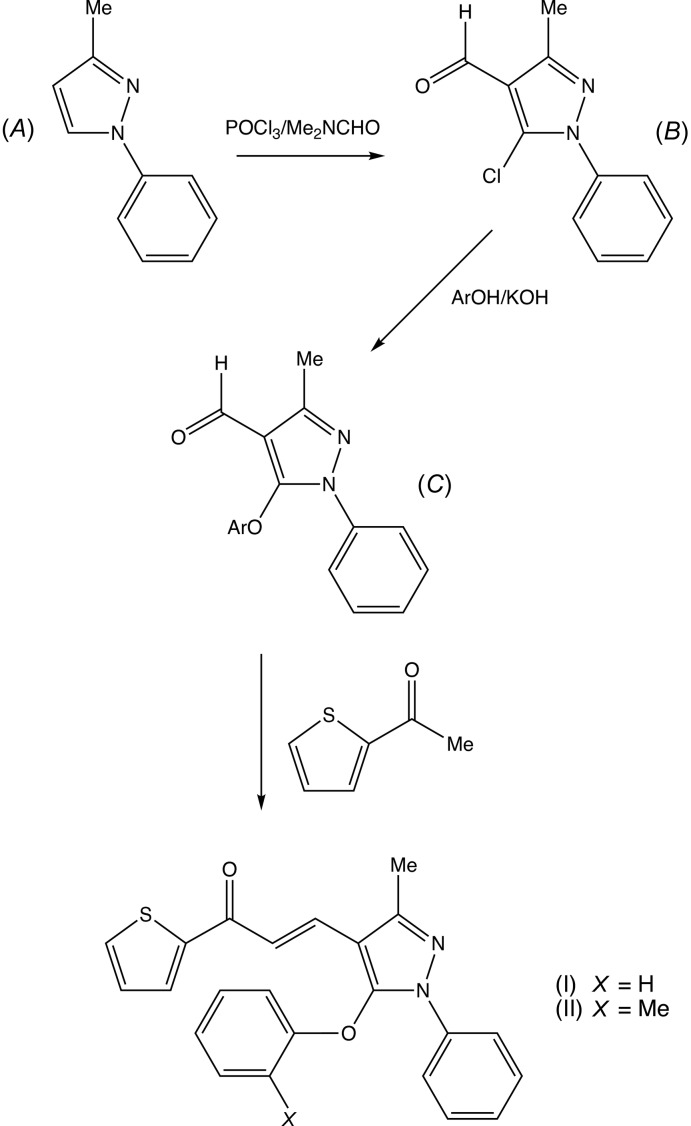
The synthetic route to compounds (I) and (II).

Compounds (I) and (II) were prepared using a three-step procedure, starting from the readily accessible 3-methyl-1-phenyl-1*H*-pyrazole (*A*) (see Fig. 4 ▸), which was converted to the corresponding 5-chloro-4-carbaldehyde (*B*) under Vilsmeier–Haack conditions, followed by nucleophilic substitution (Asma *et al.*, 2017 ▸) to provide the 5-ar­yloxy inter­mediates (*C*). Condensation with 2-acetyl­ethio­phene then gave the products (I) and (II) in yields of 86% and 84%, respectively. Thus the appropriate 3-methyl-5-ar­yloxy-1-phenyl-1*H*-pyrazole 4-carb­aldehydes (Asma *et al.*, 2017 ▸) [1.7 mmol; 445 mg for (I), or 469 mg for (II)] and 2-acetyl thio­phene (1.7 mmol, 214 mg) were dissolved in ethanol (20 ml) at 273 K; a solution of potassium hydroxide (2.1 mmol, 112 mg) in ethanol (5 ml) was then added dropwise, and the resulting mixtures were then stirred for 4 h. When the reactions were complete, as judged by thin-layer chromatography, the resulting solid products were collected by filtration, washed with water, dried in air and then recrystallized from ethanol–di­methyl­formamide (9:1, *v*/*v*), to give crystals suitable for single-crystal X-ray diffraction. Compound (I). Yield 86%, m.p. 425–427 K. IR (cm^−1^) 1667 (C=O), 1591 (C=N). Analysis: found C 71.5, H 4.7, N 7.2%: C_23_H_18_N_2_O_2_S requires C 71.5, H 4.7, N 7.3%. Compound (II). Yield 84%, m.p. 401–405 K. IR (cm^−1^) 1671 (C=O), 1564 (C=N). Analysis: found C 72.0, H 5.1, N 7.1%: C_24_H_20_N_2_O_2_S requires C 72.0, H 5.0, N 7.0%.

## Refinement   

Crystal data, data collection and structure refinement details are summarized In Table 3 ▸. In both compounds, the thienyl unit was disordered over two sets of atomic sites having unequal occupancies. In each case, the bonded distances and the 1,3 non-bonded distances in the minor disorder component were restrained to be the similar to the equivalent distances in the major disorder component, subject to s.u. values of 0.01 Å and 0.02° for bonds and angles, respectively, and the anisotropic displacement parameters for pairs of partial-occupancy atoms occupying essentially the same physical space were constrained to be equal. All H atoms, apart from those in the minor disorder components were located in difference maps, and then treated as riding atoms in geometrically idealized positions, with C—H distances of 0.93 Å (alkenyl, aromatic and thien­yl) or 0.96 Å (meth­yl), and with *U*_iso_(H) = *kU*_eq_(C), where *k* = 1.5 for the methyl groups, which were permitted to rotate but not to tilt, and 1.2 for all other H atoms. The H atoms in the minor disorder components were included on the same basis. Subject to these conditions, the occupancies of the disorder components refined to 0.844 (3) and 0.156 (3) in (I), and 0.883 (2) and to 0.117 (2) in (II).

## Supplementary Material

Crystal structure: contains datablock(s) global, I, II. DOI: 10.1107/S205698901901658X/zl2765sup1.cif

Structure factors: contains datablock(s) I. DOI: 10.1107/S205698901901658X/zl2765Isup2.hkl

Structure factors: contains datablock(s) II. DOI: 10.1107/S205698901901658X/zl2765IIsup3.hkl

Supporting information file. DOI: 10.1107/S205698901901658X/zl2765Isup4.cml

Supporting information file. DOI: 10.1107/S205698901901658X/zl2765IIsup5.cml

CCDC references: 1970925, 1970924

Additional supporting information:  crystallographic information; 3D view; checkCIF report

## Figures and Tables

**Table 1:** Hydrogen-bond geometry (Å, °) for (I)

*D*—H⋯*A*	*D*—H	H⋯*A*	*D*⋯*A*	*D*—H⋯*A*
C14—H14⋯N32^i^	0.93	2.62	3.462 (9)	151
C25—H25⋯N32^i^	0.93	2.51	3.33 (5)	148
C314—H314⋯O1^ii^	0.93	2.38	3.305 (3)	175

Symmetry codes: (i) 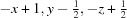; (ii) 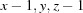.

**Table 2:** Hydrogen-bond geometry (Å, °) for (II)

*D*—H⋯*A*	*D*—H	H⋯*A*	*D*⋯*A*	*D*—H⋯*A*
C14—H14⋯N32^i^	0.93	2.55	3.483 (4)	177
C25—H25⋯N32^i^	0.93	2.69	3.47 (2)	142
C314—H314⋯O1^ii^	0.93	2.51	3.432 (3)	171

Symmetry codes: (i) 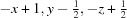; (ii) 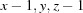.

**Table 3:** Experimental details

	(I)	(II)
Crystal data		
Chemical formula	C_23_H_18_N_2_O_2_S	C_24_H_20_N_2_O_2_S
*M*_r_	386.45	400.48
Crystal system, space group	Monoclinic, *P*2_1_/*c*	Monoclinic, *P*2_1_/*c*
Temperature (K)	296	296
*a*, *b*, *c* (Å)	9.6158 (5), 19.8846 (11), 10.3773 (6)	9.4336 (4), 20.6071 (9), 10.5866 (4)
β (°)	93.712 (2)	93.106 (2)
*V* (Å^3^)	1980.04 (19)	2055.00 (15)
*Z*	4	4
Radiation type	Mo *K*α	Mo *K*α
μ (mm^−1^)	0.18	0.18
Crystal size (mm)	0.20 × 0.20 × 0.15	0.30 × 0.20 × 0.15

Data collection		
Diffractometer	Bruker Kappa APEXII CCD	Bruker Kappa APEXII CCD
Absorption correction	Multi-scan (*SADABS*; Bruker, 2012 ▸)	Multi-scan (*SADABS*; Bruker, 2012 ▸)
*T*_min_, *T*_max_	0.941, 0.973	0.926, 0.973
No. of measured, independent and observed [*I* > 2σ(*I*)] reflections	31970, 3725, 2446	35938, 4735, 2877
*R*_int_	0.043	0.040
(sin θ/λ)_max_ (Å^−1^)	0.608	0.651

Refinement		
*R*[*F* ^2^ > 2σ(*F* ^2^)], *wR*(*F* ^2^), *S*	0.042, 0.117, 1.06	0.045, 0.142, 1.02
No. of reflections	3725	4735
No. of parameters	268	277
No. of restraints	10	10
H-atom treatment	H-atom parameters constrained	H-atom parameters constrained
Δρ_max_, Δρ_min_ (e Å^−3^)	0.20, −0.14	0.19, −0.23

Computer programs: *APEX2* (Bruker, 2012 ▸), *SAINT* (Bruker, 2017 ▸), *SHELXT* (Sheldrick, 2015*a* ▸), *SHELXL2014* (Sheldrick, 2015*b* ▸) and *PLATON* (Spek, 2009 ▸).

## supplementary crystallographic information

### 3-(3-Methyl-5-phenoxy-1-phenyl-1H-pyrazol-4-yl)-1-(thiophen-2-yl)prop-2-en-1-one (I) Crystal data

**Table:**

C_23_H_18_N_2_O_2_S	*F*(000) = 808
*M**_r_* = 386.45	*D*_x_ = 1.296 Mg m^−^^3^
Monoclinic, *P*2_1_/*c*	Mo *K*α radiation, λ = 0.71073 Å
*a* = 9.6158 (5) Å	Cell parameters from 3725 reflections
*b* = 19.8846 (11) Å	θ = 2.1–25.6°
*c* = 10.3773 (6) Å	µ = 0.18 mm^−^^1^
β = 93.712 (2)°	*T* = 296 K
*V* = 1980.04 (19) Å^3^	Block, colourless
*Z* = 4	0.20 × 0.20 × 0.15 mm

### 3-(3-Methyl-5-phenoxy-1-phenyl-1H-pyrazol-4-yl)-1-(thiophen-2-yl)prop-2-en-1-one (I) Data collection

**Table:**

Bruker Kappa APEXII CCD diffractometer	3725 independent reflections
Radiation source: fine-focus sealed tube	2446 reflections with *I* > 2σ(*I*)
Graphite monochromator	*R*_int_ = 0.043
Detector resolution: 7.3910 pixels mm^-1^	θ_max_ = 25.6°, θ_min_ = 2.1°
φ and ω scans	*h* = −10→11
Absorption correction: multi-scan (SADABS; Bruker, 2012)	*k* = −24→24
*T*_min_ = 0.941, *T*_max_ = 0.973	*l* = −12→12
31970 measured reflections	

### 3-(3-Methyl-5-phenoxy-1-phenyl-1H-pyrazol-4-yl)-1-(thiophen-2-yl)prop-2-en-1-one (I) Refinement

**Table:**

Refinement on *F*^2^	Hydrogen site location: inferred from neighbouring sites
Least-squares matrix: full	H-atom parameters constrained
*R*[*F*^2^ > 2σ(*F*^2^)] = 0.042	*w* = 1/[σ^2^(*F*_o_^2^) + (0.0467*P*)^2^ + 0.573*P*] where *P* = (*F*_o_^2^ + 2*F*_c_^2^)/3
*wR*(*F*^2^) = 0.117	(Δ/σ)_max_ < 0.001
*S* = 1.06	Δρ_max_ = 0.20 e Å^−^^3^
3725 reflections	Δρ_min_ = −0.14 e Å^−^^3^
268 parameters	Extinction correction: SHELXL, Fc^*^=kFc[1+0.001xFc^2^λ^3^/sin(2θ)]^-1/4^
10 restraints	Extinction coefficient: 0.0059 (9)
Primary atom site location: difference Fourier map	

### 3-(3-Methyl-5-phenoxy-1-phenyl-1H-pyrazol-4-yl)-1-(thiophen-2-yl)prop-2-en-1-one (I) Special details

**Table:**

Geometry. All esds (except the esd in the dihedral angle between two l.s. planes) are estimated using the full covariance matrix. The cell esds are taken into account individually in the estimation of esds in distances, angles and torsion angles; correlations between esds in cell parameters are only used when they are defined by crystal symmetry. An approximate (isotropic) treatment of cell esds is used for estimating esds involving l.s. planes.

### 3-(3-Methyl-5-phenoxy-1-phenyl-1H-pyrazol-4-yl)-1-(thiophen-2-yl)prop-2-en-1-one (I) Fractional atomic coordinates and isotropic or equivalent isotropic displacement parameters (Å^2^)

**Table:**

	*x*	*y*	*z*	*U*_iso_*/*U*_eq_	Occ. (<1)
C1	0.6952 (2)	0.38677 (11)	0.5021 (2)	0.0485 (5)	
O1	0.76942 (19)	0.43097 (8)	0.55125 (17)	0.0798 (6)	
C2	0.5895 (2)	0.40140 (11)	0.3982 (2)	0.0480 (5)	
H2	0.5376	0.3663	0.3604	0.058*	
C3	0.5660 (2)	0.46399 (10)	0.35662 (19)	0.0451 (5)	
H3	0.6175	0.4976	0.4000	0.054*	
S11	0.82626 (11)	0.29817 (5)	0.67269 (8)	0.0664 (3)	0.844 (3)
C12	0.7111 (2)	0.31696 (10)	0.5460 (2)	0.0484 (5)	0.844 (3)
C13	0.6491 (14)	0.2615 (5)	0.5017 (13)	0.0930 (17)	0.844 (3)
H13	0.5837	0.2612	0.4316	0.112*	0.844 (3)
C14	0.6904 (13)	0.2030 (2)	0.5695 (15)	0.106 (3)	0.844 (3)
H14	0.6547	0.1605	0.5506	0.128*	0.844 (3)
C15	0.7865 (9)	0.2158 (3)	0.6639 (9)	0.080 (2)	0.844 (3)
H15	0.8270	0.1831	0.7185	0.096*	0.844 (3)
S21	0.614 (2)	0.2534 (7)	0.484 (2)	0.0930 (17)	0.156 (3)
C22	0.7111 (2)	0.31696 (10)	0.5460 (2)	0.0484 (5)	0.156 (3)
C23	0.813 (2)	0.2935 (10)	0.626 (2)	0.0664 (3)	0.156 (3)
H23	0.8837	0.3205	0.6635	0.080*	0.156 (3)
C24	0.805 (6)	0.2239 (12)	0.647 (6)	0.080 (2)	0.156 (3)
H24	0.8581	0.2009	0.7111	0.096*	0.156 (3)
C25	0.713 (8)	0.1950 (8)	0.564 (9)	0.106 (3)	0.156 (3)
H25	0.7042	0.1488	0.5511	0.128*	0.156 (3)
N31	0.31385 (16)	0.49026 (8)	0.08717 (15)	0.0417 (4)	
N32	0.36763 (17)	0.55440 (8)	0.09923 (17)	0.0474 (4)	
C33	0.4603 (2)	0.55130 (10)	0.1990 (2)	0.0444 (5)	
C34	0.4706 (2)	0.48590 (10)	0.25279 (19)	0.0412 (5)	
C35	0.3759 (2)	0.44920 (9)	0.17696 (18)	0.0397 (5)	
C311	0.2118 (2)	0.47606 (10)	−0.01477 (19)	0.0400 (5)	
C312	0.1122 (2)	0.42704 (10)	−0.0019 (2)	0.0480 (5)	
H312	0.1113	0.4020	0.0737	0.058*	
C313	0.0143 (2)	0.41552 (11)	−0.1021 (2)	0.0555 (6)	
H313	−0.0532	0.3826	−0.0937	0.067*	
C314	0.0151 (2)	0.45223 (12)	−0.2146 (2)	0.0609 (7)	
H314	−0.0508	0.4439	−0.2822	0.073*	
C315	0.1141 (3)	0.50138 (13)	−0.2260 (2)	0.0596 (6)	
H315	0.1146	0.5266	−0.3015	0.071*	
C316	0.2127 (2)	0.51353 (11)	−0.1264 (2)	0.0499 (5)	
H316	0.2794	0.5468	−0.1345	0.060*	
C331	0.5426 (2)	0.61231 (11)	0.2386 (2)	0.0609 (6)	
H31A	0.6353	0.6082	0.2102	0.091*	
H31B	0.5468	0.6165	0.3309	0.091*	
H31C	0.4987	0.6514	0.2001	0.091*	
O35	0.35064 (14)	0.38210 (6)	0.17345 (13)	0.0458 (4)	
C351	0.2722 (2)	0.35338 (10)	0.2680 (2)	0.0445 (5)	
C352	0.2634 (3)	0.28476 (12)	0.2623 (3)	0.0687 (7)	
H352	0.3095	0.2609	0.2009	0.082*	
C353	0.1856 (3)	0.25194 (15)	0.3483 (4)	0.0947 (10)	
H353	0.1786	0.2053	0.3453	0.114*	
C354	0.1183 (3)	0.28709 (17)	0.4383 (4)	0.0972 (11)	
H354	0.0656	0.2644	0.4965	0.117*	
C355	0.1281 (3)	0.35565 (16)	0.4432 (3)	0.0854 (9)	
H355	0.0820	0.3793	0.5049	0.102*	
C356	0.2065 (2)	0.39028 (12)	0.3568 (2)	0.0603 (6)	
H356	0.2138	0.4369	0.3594	0.072*	

### 3-(3-Methyl-5-phenoxy-1-phenyl-1H-pyrazol-4-yl)-1-(thiophen-2-yl)prop-2-en-1-one (I) Atomic displacement parameters (Å^2^)

**Table:**

	*U*^11^	*U*^22^	*U*^33^	*U*^12^	*U*^13^	*U*^23^
C1	0.0522 (13)	0.0469 (13)	0.0450 (12)	−0.0042 (10)	−0.0060 (10)	0.0003 (10)
O1	0.0919 (13)	0.0535 (10)	0.0872 (13)	−0.0176 (9)	−0.0475 (10)	0.0094 (9)
C2	0.0493 (12)	0.0441 (12)	0.0491 (12)	−0.0035 (10)	−0.0085 (10)	−0.0002 (10)
C3	0.0465 (12)	0.0443 (12)	0.0437 (12)	−0.0051 (9)	−0.0022 (10)	−0.0015 (9)
S11	0.0811 (6)	0.0537 (5)	0.0608 (6)	−0.0027 (4)	−0.0243 (5)	0.0092 (4)
C12	0.0550 (13)	0.0451 (13)	0.0442 (12)	0.0012 (10)	−0.0038 (10)	−0.0002 (10)
C13	0.138 (7)	0.048 (2)	0.086 (4)	−0.017 (2)	−0.050 (3)	−0.004 (2)
C14	0.167 (6)	0.0398 (17)	0.104 (3)	−0.009 (3)	−0.050 (5)	0.002 (3)
C15	0.113 (4)	0.055 (2)	0.070 (4)	0.007 (2)	−0.016 (3)	0.0186 (17)
S21	0.138 (7)	0.048 (2)	0.086 (4)	−0.017 (2)	−0.050 (3)	−0.004 (2)
C22	0.0550 (13)	0.0451 (13)	0.0442 (12)	0.0012 (10)	−0.0038 (10)	−0.0002 (10)
C23	0.0811 (6)	0.0537 (5)	0.0608 (6)	−0.0027 (4)	−0.0243 (5)	0.0092 (4)
C24	0.113 (4)	0.055 (2)	0.070 (4)	0.007 (2)	−0.016 (3)	0.0186 (17)
C25	0.167 (6)	0.0398 (17)	0.104 (3)	−0.009 (3)	−0.050 (5)	0.002 (3)
N31	0.0402 (9)	0.0369 (9)	0.0473 (10)	0.0007 (7)	−0.0034 (8)	0.0033 (8)
N32	0.0489 (10)	0.0350 (10)	0.0571 (11)	−0.0031 (8)	−0.0054 (9)	0.0051 (8)
C33	0.0428 (11)	0.0390 (12)	0.0509 (13)	−0.0024 (9)	−0.0002 (10)	0.0011 (10)
C34	0.0393 (11)	0.0401 (11)	0.0437 (11)	−0.0002 (9)	−0.0002 (9)	0.0016 (9)
C35	0.0393 (11)	0.0345 (11)	0.0450 (11)	0.0012 (9)	0.0016 (9)	0.0022 (9)
C311	0.0372 (11)	0.0397 (11)	0.0425 (11)	0.0050 (9)	−0.0028 (9)	−0.0025 (9)
C312	0.0471 (12)	0.0425 (12)	0.0534 (13)	0.0040 (10)	−0.0045 (10)	−0.0020 (10)
C313	0.0485 (13)	0.0508 (14)	0.0655 (15)	0.0010 (11)	−0.0082 (11)	−0.0108 (12)
C314	0.0554 (14)	0.0677 (16)	0.0570 (15)	0.0127 (13)	−0.0149 (11)	−0.0184 (13)
C315	0.0631 (15)	0.0706 (16)	0.0441 (13)	0.0090 (13)	−0.0037 (12)	0.0026 (11)
C316	0.0473 (12)	0.0552 (14)	0.0470 (13)	0.0025 (10)	0.0016 (10)	0.0025 (10)
C331	0.0622 (15)	0.0451 (13)	0.0738 (16)	−0.0077 (11)	−0.0082 (12)	0.0016 (12)
O35	0.0525 (9)	0.0332 (8)	0.0515 (9)	−0.0010 (6)	0.0036 (7)	0.0004 (6)
C351	0.0411 (11)	0.0398 (12)	0.0515 (13)	−0.0025 (9)	−0.0047 (10)	0.0105 (10)
C352	0.0714 (16)	0.0420 (14)	0.093 (2)	−0.0053 (12)	0.0095 (15)	0.0076 (13)
C353	0.094 (2)	0.0543 (17)	0.138 (3)	−0.0114 (16)	0.023 (2)	0.0266 (19)
C354	0.093 (2)	0.087 (2)	0.115 (3)	−0.0112 (18)	0.027 (2)	0.046 (2)
C355	0.086 (2)	0.095 (2)	0.0777 (19)	−0.0017 (17)	0.0287 (16)	0.0142 (17)
C356	0.0628 (15)	0.0546 (14)	0.0640 (15)	−0.0025 (12)	0.0093 (12)	0.0034 (12)

### 3-(3-Methyl-5-phenoxy-1-phenyl-1H-pyrazol-4-yl)-1-(thiophen-2-yl)prop-2-en-1-one (I) Geometric parameters (Å, º)

**Table:**

C1—O1	1.223 (2)	C35—O35	1.356 (2)
C1—C2	1.462 (3)	C311—C316	1.378 (3)
C1—C12	1.466 (3)	C311—C312	1.379 (3)
C2—C3	1.332 (3)	C312—C313	1.376 (3)
C2—H2	0.9300	C312—H312	0.9300
C3—C34	1.437 (3)	C313—C314	1.377 (3)
C3—H3	0.9300	C313—H313	0.9300
S11—C15	1.684 (5)	C314—C315	1.375 (3)
S11—C12	1.705 (2)	C314—H314	0.9300
C12—C13	1.322 (7)	C315—C316	1.378 (3)
C13—C14	1.402 (10)	C315—H315	0.9300
C13—H13	0.9300	C316—H316	0.9300
C14—C15	1.327 (5)	C331—H31A	0.9600
C14—H14	0.9300	C331—H31B	0.9600
C15—H15	0.9300	C331—H31C	0.9600
S21—C25	1.685 (12)	O35—C351	1.398 (2)
C23—C24	1.404 (14)	C351—C356	1.365 (3)
C23—H23	0.9300	C351—C352	1.368 (3)
C24—C25	1.329 (9)	C352—C353	1.366 (4)
C24—H24	0.9300	C352—H352	0.9300
C25—H25	0.9300	C353—C354	1.364 (4)
N31—C35	1.349 (2)	C353—H353	0.9300
N31—N32	1.379 (2)	C354—C355	1.367 (4)
N31—C311	1.424 (2)	C354—H354	0.9300
N32—C33	1.323 (3)	C355—C356	1.391 (3)
C33—C34	1.416 (3)	C355—H355	0.9300
C33—C331	1.492 (3)	C356—H356	0.9300
C34—C35	1.374 (3)		
			
O1—C1—C2	121.57 (19)	C316—C311—N31	118.45 (18)
O1—C1—C12	120.34 (19)	C312—C311—N31	121.18 (18)
C2—C1—C12	118.09 (18)	C313—C312—C311	119.4 (2)
C3—C2—C1	121.35 (19)	C313—C312—H312	120.3
C3—C2—H2	119.3	C311—C312—H312	120.3
C1—C2—H2	119.3	C312—C313—C314	120.7 (2)
C2—C3—C34	127.83 (19)	C312—C313—H313	119.6
C2—C3—H3	116.1	C314—C313—H313	119.6
C34—C3—H3	116.1	C315—C314—C313	119.5 (2)
C15—S11—C12	92.15 (19)	C315—C314—H314	120.3
C13—C12—C1	130.3 (5)	C313—C314—H314	120.3
C13—C12—S11	110.0 (5)	C314—C315—C316	120.4 (2)
C1—C12—S11	119.77 (15)	C314—C315—H315	119.8
C12—C13—C14	114.2 (6)	C316—C315—H315	119.8
C12—C13—H13	122.9	C315—C316—C311	119.7 (2)
C14—C13—H13	122.9	C315—C316—H316	120.2
C15—C14—C13	111.9 (4)	C311—C316—H316	120.2
C15—C14—H14	124.0	C33—C331—H31A	109.5
C13—C14—H14	124.0	C33—C331—H31B	109.5
C14—C15—S11	111.7 (4)	H31A—C331—H31B	109.5
C14—C15—H15	124.1	C33—C331—H31C	109.5
S11—C15—H15	124.1	H31A—C331—H31C	109.5
C24—C23—H23	123.3	H31B—C331—H31C	109.5
C25—C24—C23	111.4 (14)	C35—O35—C351	119.13 (15)
C25—C24—H24	124.3	C356—C351—C352	122.3 (2)
C23—C24—H24	124.3	C356—C351—O35	123.30 (19)
C24—C25—S21	110.8 (12)	C352—C351—O35	114.3 (2)
C24—C25—H25	124.6	C353—C352—C351	118.9 (3)
S21—C25—H25	124.6	C353—C352—H352	120.5
C35—N31—N32	110.54 (15)	C351—C352—H352	120.5
C35—N31—C311	130.24 (16)	C354—C353—C352	120.4 (3)
N32—N31—C311	119.16 (15)	C354—C353—H353	119.8
C33—N32—N31	104.92 (15)	C352—C353—H353	119.8
N32—C33—C34	112.18 (17)	C353—C354—C355	120.1 (3)
N32—C33—C331	119.74 (18)	C353—C354—H354	119.9
C34—C33—C331	128.03 (19)	C355—C354—H354	119.9
C35—C34—C33	103.60 (17)	C354—C355—C356	120.6 (3)
C35—C34—C3	129.46 (18)	C354—C355—H355	119.7
C33—C34—C3	126.81 (18)	C356—C355—H355	119.7
N31—C35—O35	120.45 (17)	C351—C356—C355	117.6 (2)
N31—C35—C34	108.73 (17)	C351—C356—H356	121.2
O35—C35—C34	130.44 (17)	C355—C356—H356	121.2
C316—C311—C312	120.35 (19)		
			
O1—C1—C2—C3	−2.8 (3)	C33—C34—C35—N31	−0.8 (2)
C12—C1—C2—C3	177.6 (2)	C3—C34—C35—N31	−176.83 (19)
C1—C2—C3—C34	177.2 (2)	C33—C34—C35—O35	172.0 (2)
O1—C1—C12—C13	−175.3 (10)	C3—C34—C35—O35	−4.1 (4)
C2—C1—C12—C13	4.3 (11)	C35—N31—C311—C316	−148.8 (2)
O1—C1—C12—S11	4.0 (3)	N32—N31—C311—C316	27.9 (3)
C2—C1—C12—S11	−176.40 (16)	C35—N31—C311—C312	32.6 (3)
C15—S11—C12—C13	−0.6 (9)	N32—N31—C311—C312	−150.69 (18)
C15—S11—C12—C1	180.0 (4)	C316—C311—C312—C313	0.5 (3)
C1—C12—C13—C14	−179.5 (10)	N31—C311—C312—C313	179.09 (18)
S11—C12—C13—C14	1.1 (17)	C311—C312—C313—C314	0.2 (3)
C12—C13—C14—C15	−1 (2)	C312—C313—C314—C315	−0.7 (3)
C13—C14—C15—S11	0.7 (17)	C313—C314—C315—C316	0.6 (3)
C12—S11—C15—C14	−0.1 (11)	C314—C315—C316—C311	0.1 (3)
C23—C24—C25—S21	−12 (9)	C312—C311—C316—C315	−0.6 (3)
C35—N31—N32—C33	−1.3 (2)	N31—C311—C316—C315	−179.25 (18)
C311—N31—N32—C33	−178.64 (17)	N31—C35—O35—C351	−109.5 (2)
N31—N32—C33—C34	0.8 (2)	C34—C35—O35—C351	78.5 (3)
N31—N32—C33—C331	178.55 (18)	C35—O35—C351—C356	7.1 (3)
N32—C33—C34—C35	0.0 (2)	C35—O35—C351—C352	−174.51 (18)
C331—C33—C34—C35	−177.6 (2)	C356—C351—C352—C353	0.2 (4)
N32—C33—C34—C3	176.16 (19)	O35—C351—C352—C353	−178.2 (2)
C331—C33—C34—C3	−1.4 (4)	C351—C352—C353—C354	−0.1 (5)
C2—C3—C34—C35	4.2 (4)	C352—C353—C354—C355	0.0 (5)
C2—C3—C34—C33	−171.0 (2)	C353—C354—C355—C356	0.0 (5)
N32—N31—C35—O35	−172.30 (16)	C352—C351—C356—C355	−0.2 (4)
C311—N31—C35—O35	4.7 (3)	O35—C351—C356—C355	178.1 (2)
N32—N31—C35—C34	1.3 (2)	C354—C355—C356—C351	0.0 (4)
C311—N31—C35—C34	178.29 (18)		

### 3-(3-Methyl-5-phenoxy-1-phenyl-1H-pyrazol-4-yl)-1-(thiophen-2-yl)prop-2-en-1-one (I) Hydrogen-bond geometry (Å, º)

**Table:**

*D*—H···*A*	*D*—H	H···*A*	*D*···*A*	*D*—H···*A*
C14—H14···N32^i^	0.93	2.62	3.462 (9)	151
C25—H25···N32^i^	0.93	2.51	3.33 (5)	148
C314—H314···O1^ii^	0.93	2.38	3.305 (3)	175

Symmetry codes: (i) −*x*+1, *y*−1/2, −*z*+1/2; (ii) *x*−1, *y*, *z*−1.

### 3-[3-Methyl-5-(2-methylphenoxy)-1-phenyl-1H-pyrazol-4-yl]-1-(thiophen-2-yl)prop-2-en-1-one (II) Crystal data

**Table:**

C_24_H_20_N_2_O_2_S	*F*(000) = 840
*M**_r_* = 400.48	*D*_x_ = 1.294 Mg m^−^^3^
Monoclinic, *P*2_1_/*c*	Mo *K*α radiation, λ = 0.71073 Å
*a* = 9.4336 (4) Å	Cell parameters from 5216 reflections
*b* = 20.6071 (9) Å	θ = 2.0–28.6°
*c* = 10.5866 (4) Å	µ = 0.18 mm^−^^1^
β = 93.106 (2)°	*T* = 296 K
*V* = 2055.00 (15) Å^3^	Block, colourless
*Z* = 4	0.30 × 0.20 × 0.15 mm

### 3-[3-Methyl-5-(2-methylphenoxy)-1-phenyl-1H-pyrazol-4-yl]-1-(thiophen-2-yl)prop-2-en-1-one (II) Data collection

**Table:**

Bruker Kappa APEXII CCD diffractometer	4735 independent reflections
Radiation source: fine-focus sealed tube	2877 reflections with *I* > 2σ(*I*)
Graphite monochromator	*R*_int_ = 0.040
Detector resolution: 7.3910 pixels mm^-1^	θ_max_ = 27.6°, θ_min_ = 2.0°
φ and ω scans	*h* = −12→11
Absorption correction: multi-scan (SADABS; Bruker, 2012)	*k* = −26→26
*T*_min_ = 0.926, *T*_max_ = 0.973	*l* = −13→11
35938 measured reflections	

### 3-[3-Methyl-5-(2-methylphenoxy)-1-phenyl-1H-pyrazol-4-yl]-1-(thiophen-2-yl)prop-2-en-1-one (II) Refinement

**Table:**

Refinement on *F*^2^	Primary atom site location: difference Fourier map
Least-squares matrix: full	Hydrogen site location: inferred from neighbouring sites
*R*[*F*^2^ > 2σ(*F*^2^)] = 0.045	H-atom parameters constrained
*wR*(*F*^2^) = 0.142	*w* = 1/[σ^2^(*F*_o_^2^) + (0.0624*P*)^2^ + 0.5761*P*] where *P* = (*F*_o_^2^ + 2*F*_c_^2^)/3
*S* = 1.02	(Δ/σ)_max_ < 0.001
4735 reflections	Δρ_max_ = 0.19 e Å^−^^3^
277 parameters	Δρ_min_ = −0.23 e Å^−^^3^
10 restraints	

### 3-[3-Methyl-5-(2-methylphenoxy)-1-phenyl-1H-pyrazol-4-yl]-1-(thiophen-2-yl)prop-2-en-1-one (II) Special details

**Table:**

Geometry. All esds (except the esd in the dihedral angle between two l.s. planes) are estimated using the full covariance matrix. The cell esds are taken into account individually in the estimation of esds in distances, angles and torsion angles; correlations between esds in cell parameters are only used when they are defined by crystal symmetry. An approximate (isotropic) treatment of cell esds is used for estimating esds involving l.s. planes.

### 3-[3-Methyl-5-(2-methylphenoxy)-1-phenyl-1H-pyrazol-4-yl]-1-(thiophen-2-yl)prop-2-en-1-one (II) Fractional atomic coordinates and isotropic or equivalent isotropic displacement parameters (Å^2^)

**Table:**

	*x*	*y*	*z*	*U*_iso_*/*U*_eq_	Occ. (<1)
C1	0.6834 (2)	0.38495 (10)	0.49061 (18)	0.0484 (5)	
O1	0.75006 (19)	0.42752 (8)	0.54834 (16)	0.0747 (5)	
C2	0.5798 (2)	0.39981 (10)	0.38620 (18)	0.0484 (5)	
H2	0.5302	0.3661	0.3455	0.058*	
C3	0.5555 (2)	0.46061 (10)	0.34871 (18)	0.0464 (5)	
H3	0.6047	0.4926	0.3949	0.056*	
S11	0.81442 (11)	0.29803 (4)	0.65506 (8)	0.0669 (3)	0.883 (2)
C12	0.7062 (2)	0.31666 (10)	0.52477 (17)	0.0456 (5)	0.883 (2)
C13	0.6609 (8)	0.2617 (3)	0.4682 (5)	0.0603 (9)	0.883 (2)
H13	0.6001	0.2616	0.3961	0.072*	0.883 (2)
C14	0.7118 (5)	0.20428 (17)	0.5259 (4)	0.0643 (12)	0.883 (2)
H14	0.6912	0.1627	0.4961	0.077*	0.883 (2)
C15	0.7941 (9)	0.21758 (17)	0.6295 (6)	0.0671 (16)	0.883 (2)
H15	0.8357	0.1859	0.6819	0.080*	0.883 (2)
S21	0.6339 (18)	0.2553 (6)	0.4380 (13)	0.0603 (9)	0.117 (2)
C22	0.7062 (2)	0.31666 (10)	0.52477 (17)	0.0456 (5)	0.117 (2)
C23	0.784 (3)	0.2933 (10)	0.623 (2)	0.0669 (3)	0.117 (2)
H23	0.8396	0.3195	0.6777	0.080*	0.117 (2)
C24	0.775 (8)	0.2255 (11)	0.638 (5)	0.0671 (16)	0.117 (2)
H24	0.8252	0.2018	0.7011	0.080*	0.117 (2)
C25	0.685 (5)	0.2001 (8)	0.550 (3)	0.0643 (12)	0.117 (2)
H25	0.6544	0.1573	0.5494	0.077*	0.117 (2)
N31	0.30777 (16)	0.48865 (7)	0.08120 (15)	0.0431 (4)	
N32	0.36119 (18)	0.55036 (8)	0.09699 (16)	0.0492 (4)	
C33	0.4529 (2)	0.54660 (9)	0.19571 (19)	0.0469 (5)	
C34	0.4627 (2)	0.48304 (9)	0.24567 (18)	0.0430 (4)	
C35	0.36867 (19)	0.44824 (9)	0.16871 (17)	0.0405 (4)	
C311	0.20793 (19)	0.47603 (9)	−0.02174 (18)	0.0419 (4)	
C312	0.1090 (2)	0.42664 (10)	−0.0158 (2)	0.0495 (5)	
H312	0.1062	0.4009	0.0562	0.059*	
C313	0.0145 (2)	0.41597 (11)	−0.1180 (2)	0.0580 (6)	
H313	−0.0515	0.3826	−0.1147	0.070*	
C314	0.0169 (2)	0.45393 (12)	−0.2241 (2)	0.0624 (6)	
H314	−0.0469	0.4462	−0.2926	0.075*	
C315	0.1140 (2)	0.50340 (13)	−0.2289 (2)	0.0621 (6)	
H315	0.1154	0.5294	−0.3006	0.075*	
C316	0.2098 (2)	0.51483 (11)	−0.12766 (19)	0.0506 (5)	
H316	0.2751	0.5485	−0.1311	0.061*	
C331	0.5357 (3)	0.60493 (10)	0.2386 (2)	0.0625 (6)	
H31A	0.6318	0.6008	0.2142	0.094*	
H31B	0.5349	0.6085	0.3290	0.094*	
H31C	0.4936	0.6430	0.2003	0.094*	
O35	0.34553 (14)	0.38351 (6)	0.16246 (12)	0.0470 (3)	
C351	0.2552 (2)	0.35450 (10)	0.24627 (19)	0.0488 (5)	
C352	0.2429 (2)	0.28766 (11)	0.2301 (2)	0.0614 (6)	
C353	0.1516 (3)	0.25613 (15)	0.3056 (3)	0.0874 (10)	
H353	0.1404	0.2114	0.2977	0.105*	
C354	0.0766 (3)	0.28876 (19)	0.3921 (3)	0.0949 (11)	
H354	0.0143	0.2661	0.4410	0.114*	
C355	0.0918 (3)	0.35466 (17)	0.4081 (3)	0.0862 (9)	
H355	0.0411	0.3763	0.4681	0.103*	
C356	0.1842 (2)	0.38895 (13)	0.3332 (2)	0.0656 (6)	
H356	0.1968	0.4335	0.3424	0.079*	
C357	0.3278 (3)	0.25326 (12)	0.1355 (3)	0.0832 (9)	
H35A	0.4265	0.2546	0.1625	0.125*	
H35B	0.3141	0.2742	0.0547	0.125*	
H35C	0.2971	0.2089	0.1285	0.125*	

### 3-[3-Methyl-5-(2-methylphenoxy)-1-phenyl-1H-pyrazol-4-yl]-1-(thiophen-2-yl)prop-2-en-1-one (II) Atomic displacement parameters (Å^2^)

**Table:**

	*U*^11^	*U*^22^	*U*^33^	*U*^12^	*U*^13^	*U*^23^
C1	0.0519 (12)	0.0483 (12)	0.0442 (11)	−0.0032 (10)	−0.0045 (9)	0.0023 (9)
O1	0.0920 (12)	0.0541 (9)	0.0734 (10)	−0.0092 (9)	−0.0381 (9)	0.0023 (8)
C2	0.0481 (11)	0.0482 (12)	0.0476 (11)	−0.0023 (9)	−0.0087 (9)	0.0021 (9)
C3	0.0468 (11)	0.0481 (12)	0.0437 (11)	−0.0023 (9)	−0.0039 (9)	−0.0007 (9)
S11	0.0885 (6)	0.0555 (4)	0.0533 (5)	0.0004 (4)	−0.0283 (3)	0.0057 (3)
C12	0.0471 (11)	0.0492 (11)	0.0399 (10)	0.0002 (9)	−0.0036 (8)	0.0023 (9)
C13	0.071 (3)	0.0556 (17)	0.051 (3)	−0.0026 (16)	−0.0199 (18)	−0.0051 (17)
C14	0.079 (3)	0.0449 (13)	0.068 (2)	−0.0014 (13)	−0.004 (2)	−0.0002 (12)
C15	0.081 (3)	0.0560 (16)	0.0624 (19)	0.0084 (19)	−0.008 (2)	0.0112 (16)
S21	0.071 (3)	0.0556 (17)	0.051 (3)	−0.0026 (16)	−0.0199 (18)	−0.0051 (17)
C22	0.0471 (11)	0.0492 (11)	0.0399 (10)	0.0002 (9)	−0.0036 (8)	0.0023 (9)
C23	0.0885 (6)	0.0555 (4)	0.0533 (5)	0.0004 (4)	−0.0283 (3)	0.0057 (3)
C24	0.081 (3)	0.0560 (16)	0.0624 (19)	0.0084 (19)	−0.008 (2)	0.0112 (16)
C25	0.079 (3)	0.0449 (13)	0.068 (2)	−0.0014 (13)	−0.004 (2)	−0.0002 (12)
N31	0.0448 (9)	0.0350 (8)	0.0485 (9)	−0.0001 (7)	−0.0054 (7)	0.0033 (7)
N32	0.0528 (10)	0.0345 (9)	0.0591 (10)	−0.0033 (7)	−0.0083 (8)	0.0040 (8)
C33	0.0475 (11)	0.0377 (10)	0.0548 (12)	−0.0009 (9)	−0.0022 (9)	0.0005 (9)
C34	0.0422 (10)	0.0387 (10)	0.0477 (11)	0.0013 (8)	−0.0024 (8)	0.0012 (8)
C35	0.0413 (10)	0.0335 (10)	0.0467 (11)	0.0003 (8)	0.0008 (8)	0.0030 (8)
C311	0.0391 (10)	0.0402 (10)	0.0458 (11)	0.0067 (8)	−0.0027 (8)	−0.0022 (8)
C312	0.0479 (11)	0.0452 (11)	0.0546 (12)	0.0027 (9)	−0.0042 (9)	0.0008 (9)
C313	0.0506 (12)	0.0577 (13)	0.0643 (14)	0.0000 (11)	−0.0091 (10)	−0.0109 (11)
C314	0.0555 (13)	0.0760 (16)	0.0541 (14)	0.0087 (12)	−0.0130 (10)	−0.0127 (12)
C315	0.0601 (14)	0.0776 (16)	0.0479 (12)	0.0094 (12)	−0.0039 (10)	0.0072 (11)
C316	0.0470 (11)	0.0542 (13)	0.0505 (12)	0.0032 (10)	0.0008 (9)	0.0051 (10)
C331	0.0679 (15)	0.0413 (12)	0.0766 (15)	−0.0052 (10)	−0.0124 (12)	−0.0050 (11)
O35	0.0492 (8)	0.0340 (7)	0.0575 (8)	−0.0018 (6)	0.0006 (6)	0.0031 (6)
C351	0.0394 (10)	0.0485 (12)	0.0570 (12)	−0.0034 (9)	−0.0099 (9)	0.0174 (10)
C352	0.0555 (13)	0.0484 (13)	0.0770 (15)	−0.0111 (11)	−0.0283 (12)	0.0223 (12)
C353	0.0746 (18)	0.0729 (19)	0.111 (2)	−0.0286 (15)	−0.0290 (18)	0.0405 (18)
C354	0.0654 (18)	0.108 (3)	0.111 (3)	−0.0234 (18)	−0.0064 (17)	0.059 (2)
C355	0.0606 (16)	0.116 (3)	0.0831 (19)	0.0065 (16)	0.0130 (14)	0.0311 (18)
C356	0.0562 (14)	0.0687 (16)	0.0725 (15)	0.0046 (12)	0.0073 (12)	0.0193 (13)
C357	0.108 (2)	0.0412 (13)	0.097 (2)	−0.0010 (14)	−0.0277 (18)	−0.0037 (13)

### 3-[3-Methyl-5-(2-methylphenoxy)-1-phenyl-1H-pyrazol-4-yl]-1-(thiophen-2-yl)prop-2-en-1-one (II) Geometric parameters (Å, º)

**Table:**

C1—O1	1.223 (2)	C311—C316	1.378 (3)
C1—C12	1.466 (3)	C311—C312	1.385 (3)
C1—C2	1.468 (3)	C312—C313	1.382 (3)
C2—C3	1.330 (3)	C312—H312	0.9300
C2—H2	0.9300	C313—C314	1.371 (3)
C3—C34	1.437 (3)	C313—H313	0.9300
C3—H3	0.9300	C314—C315	1.373 (3)
S11—C15	1.689 (4)	C314—H314	0.9300
S11—C12	1.7146 (19)	C315—C316	1.384 (3)
C12—C13	1.340 (5)	C315—H315	0.9300
C13—C14	1.404 (6)	C316—H316	0.9300
C13—H13	0.9300	C331—H31A	0.9600
C14—C15	1.337 (3)	C331—H31B	0.9600
C14—H14	0.9300	C331—H31C	0.9600
C15—H15	0.9300	O35—C351	1.397 (2)
S21—C25	1.692 (11)	C351—C356	1.367 (3)
C23—C24	1.408 (11)	C351—C352	1.392 (3)
C23—H23	0.9300	C352—C353	1.370 (4)
C24—C25	1.340 (9)	C352—C357	1.495 (4)
C24—H24	0.9300	C353—C354	1.364 (5)
C25—H25	0.9300	C353—H353	0.9300
N31—C35	1.351 (2)	C354—C355	1.375 (4)
N31—N32	1.375 (2)	C354—H354	0.9300
N31—C311	1.425 (2)	C355—C356	1.400 (3)
N32—C33	1.322 (2)	C355—H355	0.9300
C33—C34	1.414 (3)	C356—H356	0.9300
C33—C331	1.491 (3)	C357—H35A	0.9600
C34—C35	1.373 (3)	C357—H35B	0.9600
C35—O35	1.353 (2)	C357—H35C	0.9600
			
O1—C1—C12	120.05 (18)	C313—C312—H312	120.4
O1—C1—C2	121.99 (19)	C311—C312—H312	120.4
C12—C1—C2	117.96 (17)	C314—C313—C312	120.9 (2)
C3—C2—C1	121.21 (18)	C314—C313—H313	119.6
C3—C2—H2	119.4	C312—C313—H313	119.6
C1—C2—H2	119.4	C313—C314—C315	119.6 (2)
C2—C3—C34	128.12 (19)	C313—C314—H314	120.2
C2—C3—H3	115.9	C315—C314—H314	120.2
C34—C3—H3	115.9	C314—C315—C316	120.4 (2)
C15—S11—C12	91.91 (14)	C314—C315—H315	119.8
C13—C12—C1	131.5 (3)	C316—C315—H315	119.8
C13—C12—S11	109.4 (3)	C311—C316—C315	119.7 (2)
C1—C12—S11	119.10 (15)	C311—C316—H316	120.2
C12—C13—C14	115.1 (3)	C315—C316—H316	120.2
C12—C13—H13	122.4	C33—C331—H31A	109.5
C14—C13—H13	122.4	C33—C331—H31B	109.5
C15—C14—C13	110.7 (3)	H31A—C331—H31B	109.5
C15—C14—H14	124.6	C33—C331—H31C	109.5
C13—C14—H14	124.6	H31A—C331—H31C	109.5
C14—C15—S11	112.9 (3)	H31B—C331—H31C	109.5
C14—C15—H15	123.6	C35—O35—C351	119.55 (16)
S11—C15—H15	123.6	C356—C351—C352	123.8 (2)
C24—C23—H23	122.8	C356—C351—O35	122.94 (19)
C25—C24—C23	110.3 (12)	C352—C351—O35	113.3 (2)
C25—C24—H24	124.9	C353—C352—C351	116.7 (3)
C23—C24—H24	124.9	C353—C352—C357	122.8 (3)
C24—C25—S21	112.0 (11)	C351—C352—C357	120.5 (2)
C24—C25—H25	124.0	C354—C353—C352	121.5 (3)
S21—C25—H25	124.0	C354—C353—H353	119.2
C35—N31—N32	110.33 (15)	C352—C353—H353	119.2
C35—N31—C311	130.62 (16)	C353—C354—C355	121.0 (3)
N32—N31—C311	118.98 (15)	C353—C354—H354	119.5
C33—N32—N31	105.17 (15)	C355—C354—H354	119.5
N32—C33—C34	112.14 (17)	C354—C355—C356	119.5 (3)
N32—C33—C331	120.20 (18)	C354—C355—H355	120.2
C34—C33—C331	127.61 (18)	C356—C355—H355	120.2
C35—C34—C33	103.57 (16)	C351—C356—C355	117.5 (3)
C35—C34—C3	129.00 (18)	C351—C356—H356	121.2
C33—C34—C3	127.36 (18)	C355—C356—H356	121.2
N31—C35—O35	120.86 (16)	C352—C357—H35A	109.5
N31—C35—C34	108.79 (16)	C352—C357—H35B	109.5
O35—C35—C34	129.88 (17)	H35A—C357—H35B	109.5
C316—C311—C312	120.13 (18)	C352—C357—H35C	109.5
C316—C311—N31	118.65 (18)	H35A—C357—H35C	109.5
C312—C311—N31	121.21 (17)	H35B—C357—H35C	109.5
C313—C312—C311	119.3 (2)		
			
O1—C1—C2—C3	1.0 (3)	C3—C34—C35—N31	−177.55 (18)
C12—C1—C2—C3	−178.86 (19)	C33—C34—C35—O35	171.48 (19)
C1—C2—C3—C34	177.23 (19)	C3—C34—C35—O35	−5.6 (3)
O1—C1—C12—C13	−170.9 (5)	C35—N31—C311—C316	−150.6 (2)
C2—C1—C12—C13	9.0 (6)	N32—N31—C311—C316	25.9 (3)
O1—C1—C12—S11	6.0 (3)	C35—N31—C311—C312	30.5 (3)
C2—C1—C12—S11	−174.22 (15)	N32—N31—C311—C312	−153.05 (18)
C15—S11—C12—C13	−0.3 (5)	C316—C311—C312—C313	1.3 (3)
C15—S11—C12—C1	−177.8 (4)	N31—C311—C312—C313	−179.78 (18)
C1—C12—C13—C14	176.4 (4)	C311—C312—C313—C314	−0.6 (3)
S11—C12—C13—C14	−0.6 (7)	C312—C313—C314—C315	−0.3 (3)
C12—C13—C14—C15	1.6 (9)	C313—C314—C315—C316	0.4 (3)
C13—C14—C15—S11	−1.8 (9)	C312—C311—C316—C315	−1.2 (3)
C12—S11—C15—C14	1.3 (7)	N31—C311—C316—C315	179.88 (18)
C23—C24—C25—S21	9 (8)	C314—C315—C316—C311	0.3 (3)
C35—N31—N32—C33	−0.9 (2)	N31—C35—O35—C351	−105.1 (2)
C311—N31—N32—C33	−178.00 (16)	C34—C35—O35—C351	83.7 (2)
N31—N32—C33—C34	0.6 (2)	C35—O35—C351—C356	2.2 (3)
N31—N32—C33—C331	178.19 (19)	C35—O35—C351—C352	−179.18 (16)
N32—C33—C34—C35	0.0 (2)	C356—C351—C352—C353	1.2 (3)
C331—C33—C34—C35	−177.5 (2)	O35—C351—C352—C353	−177.45 (18)
N32—C33—C34—C3	177.07 (18)	C356—C351—C352—C357	−178.1 (2)
C331—C33—C34—C3	−0.4 (4)	O35—C351—C352—C357	3.3 (3)
C2—C3—C34—C35	6.2 (4)	C351—C352—C353—C354	0.0 (4)
C2—C3—C34—C33	−170.2 (2)	C357—C352—C353—C354	179.3 (2)
N32—N31—C35—O35	−171.96 (16)	C352—C353—C354—C355	−1.0 (4)
C311—N31—C35—O35	4.7 (3)	C353—C354—C355—C356	0.9 (4)
N32—N31—C35—C34	0.9 (2)	C352—C351—C356—C355	−1.3 (3)
C311—N31—C35—C34	177.56 (18)	O35—C351—C356—C355	177.17 (19)
C33—C34—C35—N31	−0.5 (2)	C354—C355—C356—C351	0.3 (4)

### 3-[3-Methyl-5-(2-methylphenoxy)-1-phenyl-1H-pyrazol-4-yl]-1-(thiophen-2-yl)prop-2-en-1-one (II) Hydrogen-bond geometry (Å, º)

**Table:**

*D*—H···*A*	*D*—H	H···*A*	*D*···*A*	*D*—H···*A*
C14—H14···N32^i^	0.93	2.55	3.483 (4)	177
C25—H25···N32^i^	0.93	2.69	3.47 (2)	142
C314—H314···O1^ii^	0.93	2.51	3.432 (3)	171

Symmetry codes: (i) −*x*+1, *y*−1/2, −*z*+1/2; (ii) *x*−1, *y*, *z*−1.
